# A network analysis of crab metamorphosis and the hypothesis of development as a process of unfolding of an intensive complexity

**DOI:** 10.1038/s41598-021-88662-1

**Published:** 2021-05-05

**Authors:** Agustín Ostachuk

**Affiliations:** 1 Museo de La Plata (MLP), Universidad Nacional de La Plata (UNLP), Buenos Aires, Argentina; 2 Centro de Estudios de Historia de la Ciencia y la Técnica José Babini (CEJB), Universidad Nacional de San Martín (UNSAM), Buenos Aires, Argentina; 3 Laboratorio de Investigación en Ciencias Humanas (LICH-CONICET), Universidad Nacional de San Martín (UNSAM), Buenos Aires, Argentina

**Keywords:** Evolutionary developmental biology, Network topology, Complexity

## Abstract

Development has intrigued humanity since ancient times. Today, the main paradigm in developmental biology and evolutionary developmental biology (evo-devo) is the genetic program, in which development is explained by the interplay and interaction of genes, that is, by the action of gene regulatory networks (GRNs). However, it is not even clear that a GRN, no matter how complex, can be translated into a form. Therefore, the fundamental enigma of development still remains: how is a complex organism formed from a single cell? This question unfolded the historical drama and the dialectical tension between preformation and epigenesis. In order to shed light on these issues, I studied the development of crabs (infraorder Brachyura), as representative of the subphylum Crustacea, using network theory. The external morphology of the different phases of brachyuran metamorphosis were modeled as networks and their main characteristics analyzed. As one could expect, the parameters usually regarded as indicative of network complexity, such as modularity and hierarchy, increased during development. However, when more sophisticated complexity measures were tested, it was evidenced that whereas a group of complexity measures increased during development, another group decreased. This led to consider that two kinds of complexities were being measured. I called them intensive and extensive complexity. In view of these results, I propose that crab development involves a passage from an intensive to an extensive complexity. In other words, crab development can be interpreted as a process of unfolding of an intensive, preexistent complexity.

## Introduction

Development has intrigued humanity since ancient times. Aristotle wrote a whole treatise on the subject, known as *De generatione animalium*. This treatise contains perhaps one of the first discussions between preformation and epigenesis. It is generally regarded that Aristotle advocated for epigenesis, but this is half true. There, Aristotle explained development in terms of his ontological system, that is, resorting to his concepts of *eidos* (form), *hyle* (matter), *dynamis* (potentiality) and *energeia* (actuality). In this manner, the embryo was formed by the union of form and matter provided by the progenitors’ seminal fluids, and development consisted of the actualization of the potentialities of the embryo, a process governed by the *psyche*^1^. He even ventured to affirm that “in the embryo all the parts exist potentially”^2^, 740a2] and that “the embryo is already potentially an animal”^2^, 740a24]. This is consistent with my recent postulate that a *virtual preformation* is necessary to explain evolution and development^3,4^.

Today, the main paradigm in developmental biology and evolutionary developmental biology (evo-devo) is the genetic program, in which development is explained by the interplay and interaction of genes, that is, by the action of gene regulatory networks (GRNs), under the master control of *Hox* genes^5–11^. However, there are alternatives to this paradigm^12,13^. One of them is the physical program, which studies the activity and influence of physical determinants on development, determinants present in living and non-living materials (such as viscoelasticity, differential cohesivity, biochemical diffusion and oscillation), and that can act as generators of novelty in the evolutionary process, giving rise to “generic forms”^14–16^. Another alternative is the morphological program, which studies the modifications in the developmental processes of organisms throughout evolution, using powerful morphometric techniques, such as geometric morphometrics^17,18^, and explaining these changes (detected as changes in the occupation of a morphospace) using concepts such as heterochrony and allometry^19–23^.

According to the “genetic theory of morphological evolution”, that is, to the genetic program: “(1) form evolves largely by altering the expression of functionally conserved proteins; and (2) such changes largely occur through mutations in the cis-regulatory regions of mosaically pleiotropic developmental regulatory genes and of target genes within the vast regulatory networks they control”^8^. However, it is not even clear that a GRN, no matter how complex, can be translated into a form. They are different levels of biological organization. In fact, we could affirm, resorting to an Aristotelian formula, that GRNs (like matter) are *for the sake of* forms, and not the other way around. This means that GRNs are at the service of form. GRNs are configurations that are configured by form. Therefore, GRNs would not determine form nor its evolution and development.

The fundamental enigma of development still remains: how is a complex organism formed from a single cell? This question unfolded the historical drama and the dialectical tension between preformation and epigenesis. Epigenesis had the advantage of not needing to assume any already formed organ in the fertilized egg. However, it always needed to resort to some formative power that guided the developmental process^3^. August Weismann considered that epigenesis was unsustainable and that a new preformationist theory (evolutionary theory, in the terminology of those days) was needed. In this manner, he proposed the germ-plasm theory, historically regarded as the coup de grâce for the final decline of Lamarckism. Weismann proposed a segregation model of development, similar to the mosaic theory advanced by Wilhelm Roux^3,24^. This theory essentially proposed that development, and finally organismal form, was determined by the architectural organization of the germ-plasm (i.e. chromatin). Both theories were refuted by the experiments of Hans Driesch and Hans Spemann, who divided the first blastomeres of an embryo and obtained a whole embryo. Again, this was interpreted as a triumph of epigenesis. The previous disquisition leads to ask the following questions: is not the GRN theory of development a more sophisticated model of the mosaic theory of development? Is not the GRN theory of development also refuted by the experiments of Driesch and Spemann?

Consequently, there is still a need to explain how the extraordinary complexity of an organism is attained. This question may in turn lead to wonder about the origin and source of this complexity. In order to shed light on these issues, I studied the development of crabs (infraorder Brachyura), as representative of the subphylum Crustacea, using network theory. The external morphology of the different phases of brachyuran metamorphosis were modeled as networks and their main characteristics analyzed. As one could expect, the parameters usually regarded as indicative of network complexity, such as modularity and hierarchy, increased during development. However, when more sophisticated complexity measures were tested, it was evidenced that whereas a group of complexity measures increased during development, another group decreased. This led to consider that two kinds of complexities were being measured. I called them intensive and extensive complexity. In view of these results, I propose that crab development involves a passage from an intensive to an extensive complexity. In other words, crab development can be interpreted as a process of unfolding of an intensive, preexistent complexity.

## Materials and methods

### The crustacean network model

**Figure 1:**
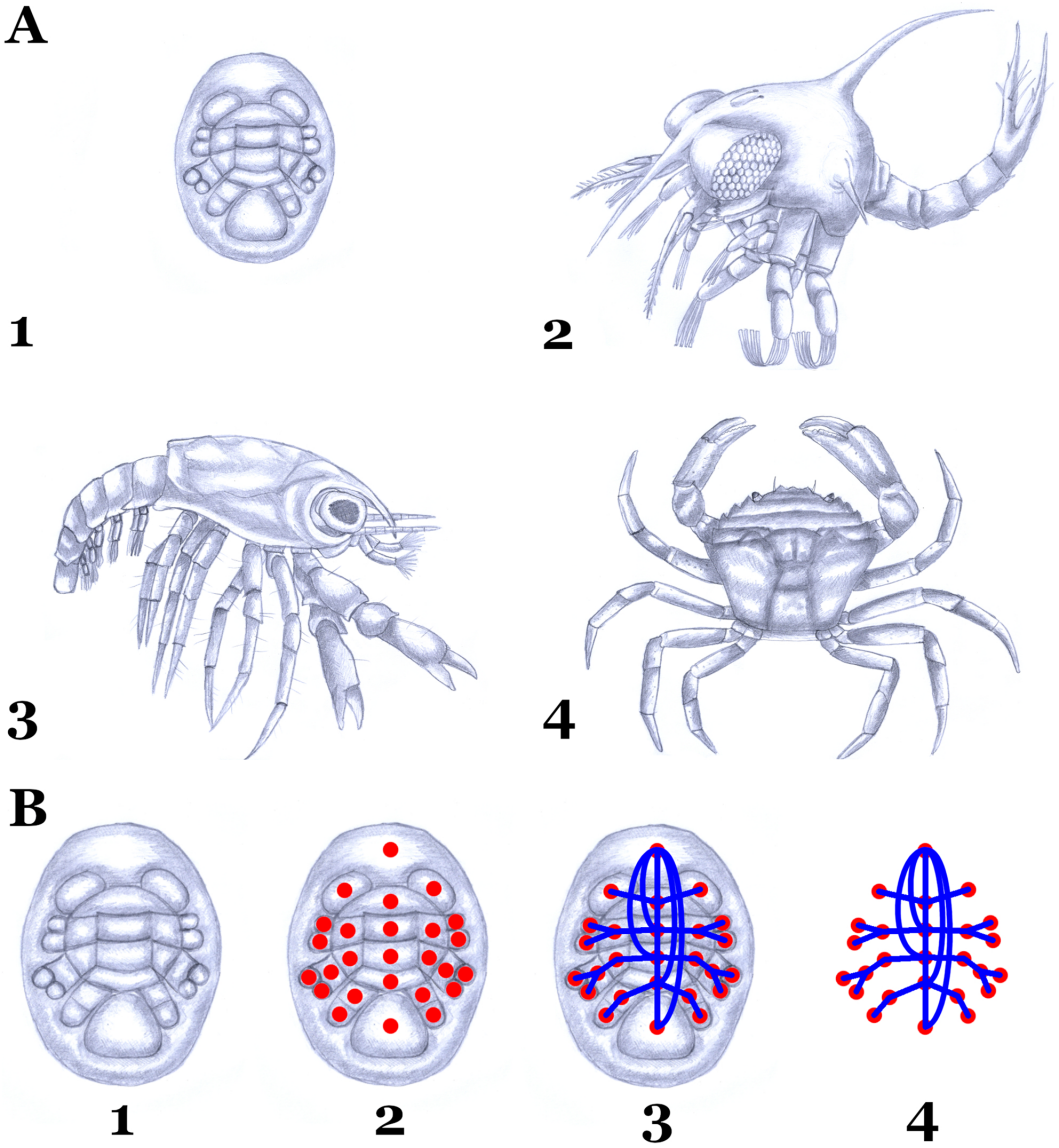
(**A**) Phases of crab metamorphosis: (1) Egg-nauplius, (2) Zoea, (3) Megalopa and (4) Crab (adult). (**B**) Stages in the construction of the crustacean complex networks: (1) detailed study of the external morphology of the animal; (2) determination of the morphological units of the animal (segments, articles, endites, exites, epipods, etc.) and their establishment as nodes; (3) determination of the physical connections between morphological units (nodes) and their establishment as links or edges; (4) the complex network is completely defined as a set of nodes connected by links or edges, which represents a model of the external morphology of the entire animal. Scientific Artwork (drawings) by Agustín Ostachuk.

In this work, I studied the development of brachyuran crabs. The analysis was based, whenever possible, on crabs of the family Portunidae (Malacostraca: Eucarida: Decapoda: Brachyura). For this purpose, the main phases of crab metamorphosis were studied (Fig. 1A): Egg-nauplius^25–27^, Zoea^28^, Megalopa^29–34^ and Crab (adult)^35–38^. The information necessary for the construction of the crustacean network models were obtained from specialized bibliography. The main references are cited above after each developmental phase. In the particular case of the egg-nauplius, the most pertinent and detailed information was from the infraorder Caridea, so the egg-nauplius network was constructed based on this information. It is known that this phase is highly conserved in the order Decapoda, so this assumption is supported by existing evidence.

Crustacean external morphology was modeled using complex networks^39–41^. As defined in our previous study^42^, morphological features identifiable and distinguishable as individual units (segments, articles, endites, exites, epipods, flagella) were abstracted as nodes, whereas physical connections between these individual units (nodes) were considered edges (Fig. 1B). Once nodes and their connections were defined, the information was coded in a square matrix $N \times N$ (adjacency matrix), where *N* was the total number of nodes of the network. Connections were coded following the binary code: 1 for its presence, 0 for its absence. The adjacency matrices generated for the different phases of crab metamorphosis are provided as Supplementary Material.

### Modularity

A module is defined as a morphological unit which parts have more connections among themselves (within the module) than to other parts outside the module^43^. Modules are considered to be the building blocks of evolutionary and developmental transformation^44^. Therefore, it would be very interesting to study the transformations of the network modular organization during the successive phases of crab development.

The identification of modules was made using multi-level optimization of modularity^45^. Community structure detection by multi-level optimization of modularity is based on the modularity measure (Q-value)^46^ and a hierarchical approach^42^.

### Hierarchical organization

#### Topological overlap

A hierarchical organization is characterized by the presence of modules that are subdivided into smaller modules^43^. A hierarchical organization is a prerequisite and a manifestation of different structural levels. A higher hierarchical structure will have more constraints and acquired compromises^47^ and will be more stable to changes, all of which is compatible with Riedl’s concept of burden^48^.

The identification of the hierarchical organization was made using topological overlap analysis^42^. The topological overlap ($O_T$) is a normalized measure of interconnectedness and relatedness that quantifies common neighbors between pairs of nodes^49^. Two nodes connected to the same nodes will have a topological overlap of 1, whereas two nodes sharing no connections will have a topological overlap of 0.

### Functionality

#### Within-module degree and participation coefficient

Nodes fulfill different roles within a given module^50^. Some of them will only be connected to other nodes within the same module, whereas others will have special roles that will make them particularly subjected to evolutionary and developmental constraints.

The study of the different roles carried out by the nodes within a given module was made by a ZP space analysis^42^. This approach is based on the idea that nodes with the same role should have similar topological properties. The position of a node within its own module and with respect to the other modules are determined by two parameters: the participation coefficient and the within-module degree^50^. Nodes with high participation coefficient (*P*) are nodes with many intermodular connections and are considered connectors. Nodes with high within-module degree (*z*) are nodes with many intramodular connections and are considered module hubs.

### Complexity measures

It is a current and ongoing debate what is a complex graph or network^51^. Real-world networks are usually more complex than random networks, due to the presence of special features such as the ones mentioned before: modular structures, hierarchical organization and different node roles. These networks typically have a medium number of edges (complete networks do not possess inner intrincate structures), so complexity measures should be high for these type of networks. They also should be normalized measures of complexity in order to be able to compare networks with different number of nodes.

There are basically three kinds of Complexity measures: Product measures, Entropy measures and Subgraph measures^42,51^. In the first group, we have Medium Articulation (*MAg*), Efficiency complexity (*Ce*)^52^ and Graph index complexity (*Cr*). In the second group, we have Offdiagonal complexity (*OdC*)^53^, Spanning tree sensitivity (*STS*) and Spanning tree sensitivity differences (*STSD*). In the third group, we have One-Edge-Deleted subgraph complexity with respect to the different number of spanning trees ($C_{1e,ST}$) and One-Edge-Deleted subgraph complexity with respect to the different spectra of the Laplacian matrix ($C_{1e,spec}$).

### Topological descriptors

Classical network descriptors, e.g. degree distribution, clustering coefficients and betweenness centrality, provide primary information about network characteristics, like hubs, clusters, etc. However, there exists a large number of more sophisticated topological network measures^54–56^. Many of these topological descriptors were developed for molecular identification and discrimination, and were not used extensively for the study of biological networks^42^. In this work, various of these measures were used for exploring their behavior in biological networks.

There are basically four kinds of topological descriptors: 1) Distance-based descriptors, 2) Other-invariants descriptors, 3) Entropy-based descriptors and 4) Eigenvalue-based descriptors^42,54–56^. In the first group, we have the Wiener index^57^, Balaban J index^58^, Compactness^59^ and Centralization^60^. In the second group, we have the Zagreb index^61^, Randić connectivity index^62^, Complexity index B^63^ and Normalized edge complexity^63^. In the third group, we have the Topological information content^64,65^, Bonchev index^66^, Bertz complexity index^67^, Radial centric information index^68^, Balaban-like information indices^69^ and Edge equality^70^. In the fourth group, we have the Estrada and Laplacian Estrada indices^55^, and Energy and Laplacian energy indices^71^.

### Network models

Network models were generated using the algorithm implemented by Viger and Latapy^72^. This method creates networks from a given degree sequence, and generates simple, connected and undirected networks. The algorithm first creates a simple undirected, possibly unconnected network. Subsequently, it performs a rewiring to obtain a connected network. Finally, a Monte-Carlo algorithm is used to randomize the network.

### Core size

A core is a group of central and densely interconnected high-degree nodes which governs the overall behaviour of a network, such as controllability, flexibility and adaptability^73^. A small core makes a network more controllable, whereas a large core makes a network more flexible and adaptable to changes^42^.

### Error and attack tolerance of complex networks

Complex networks display a high tolerance against errors. This robustness is often attributed to the redundant wiring of the network’s elements. However, error tolerance comes at a high price: these networks are extremely vulnerable to attacks^74^. This property could be exploited for the detection of vital nodes or structures that are essential for maintaining the network’s functioning (i.e. connectivity).

This behavior can be simulated and tested by the random (error) or selected (attack) removal of nodes, and assessment of the resultant loss of connectivity. Three different attack strategies were evaluated: betweenness-based attack, degree-based attack and cascading attack, where betweenness is recalculated after each node is removed. In each case, nodes were removed one by one, randomly or in the decreasing order of their betweenness centrality or degree.

### Software

Network analysis was carried out using the R programming language^75,76^. Several packages from this project were used for different purposes. The package *igraph*^77^ was used for the analysis of Network parameters and Modularity, and for the generation of Network models. The package *brainGraph*^78^ was used for ZP space analysis and the package *WGCNA*^79^ for Topological Overlap analysis. The package *QuACN*^54^ was used for Complexity analysis. The package *NetSwan*^80^ was used for Network strengths and weaknesses analysis. Plots were made using the package *ggplot2*^81^. Visual network analysis was carried out using the program *Gephi*^82^.

## Results

### Description and analysis of networks

**Figure 2:**
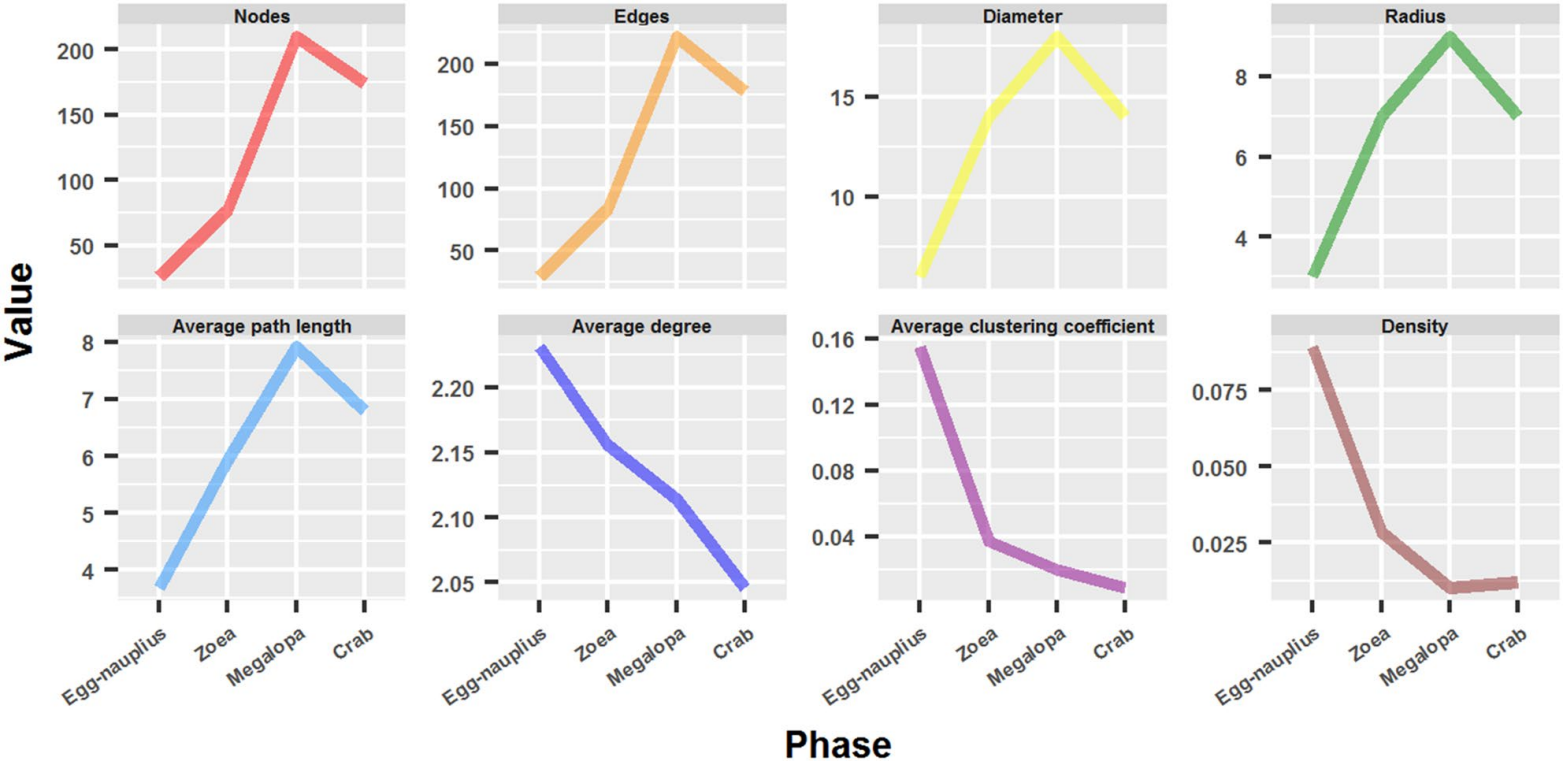
Network parameters of the different phases of crab development.

The different phases of crab metamorphosis (Egg-nauplius, Zoea, Megalopa and Adult crab) were modeled as networks. The most common network parameters are summarized in Fig. 2 and Table 1. Size and distance parameters, such as number of Nodes and Edges, Diameter, Radius and Average path length increased during crab development, with the exception of the passage from the megalopa to the adult phase where these parameters inverted their tendency and decreased. On the other hand, interconnective parameters, such as Average degree, Average clustering coefficient and Density, decreased, including the transition from megalopa to adult. The only exception to this behavior was the density of the adult crab network, which was a bit higher than in the megalopa phase.

**Table 1:** Network parameters of the different phases of crab development.

Network parameter	Egg-nauplius	Zoea	Megalopa	Crab
Nodes	26	77	210	174
Edges	29	83	222	178
Diameter	6	14	18	14
Radius	3	7	9	7
Average path length	3.689231	5.930622	7.910823	6.791841
Average degree	2.231	2.156	2.114	2.046
Average clustering coefficient	0.1547619	0.03731884	0.0199257	0.008788932
Density	0.08923077	0.02836637	0.0101162	0.01182646

### Visual analysis of crab developmental networks

**Figure 3:**
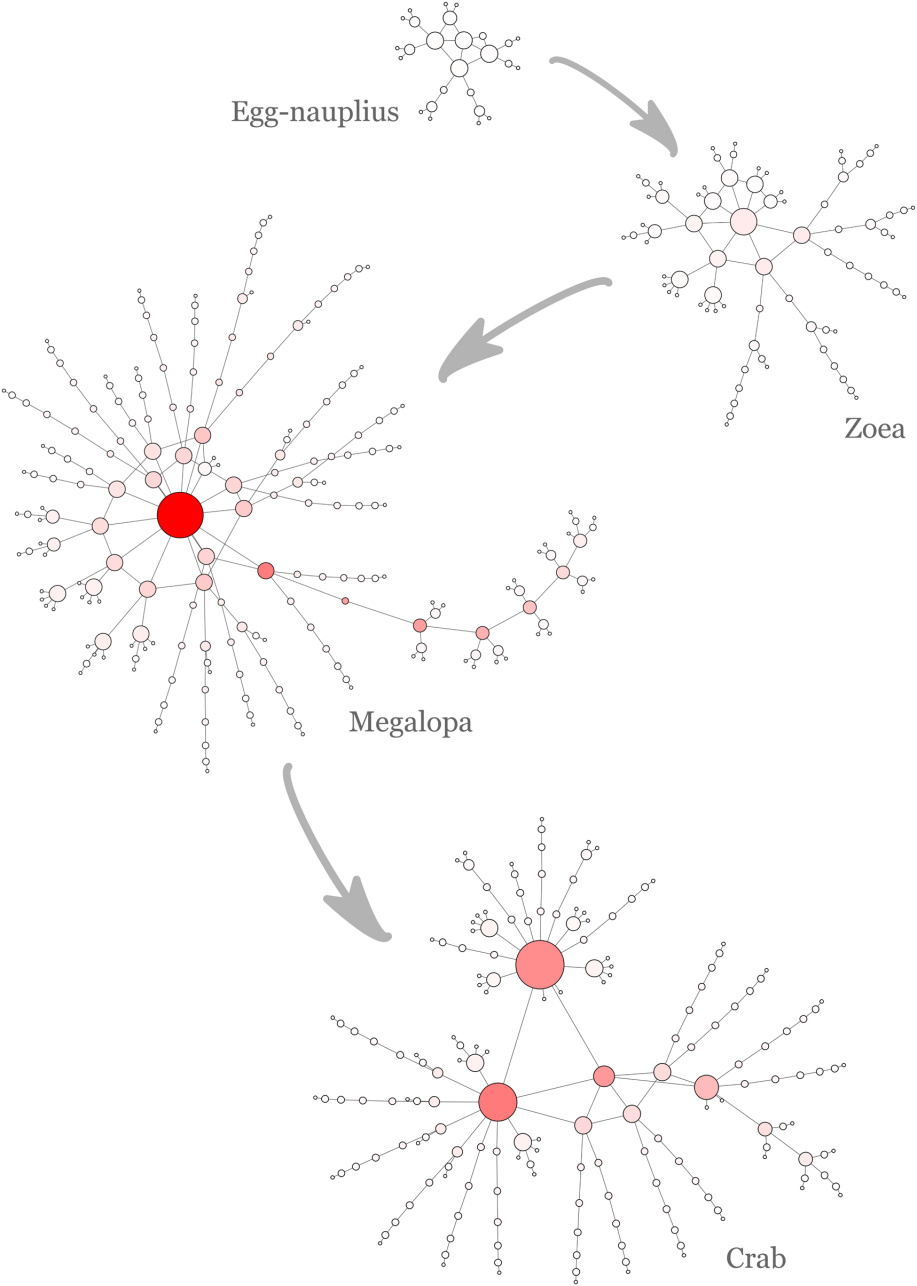
Crab developmental networks. The networks corresponding to the different phases of crab development were visually analyzed using *Gephi*. Networks were plotted using the Force Atlas layout algorithm. Node size corresponds to node degree, whereas node color intensity corresponds to node betweenness centrality. Networks were scaled so that comparisons can be made.

Networks corresponding to the different phases of crab metamorphosis were studied using the software *Gephi*. Figure 3 shows the crab developmental networks analyzed and plotted with this software. Node size represents its degree, while node color represents its betweenness centrality (the redder, the greater the betweenness centrality). Size and color were scaled in the four networks to make comparisons between them possible.

Crab metamorphosis was marked by an increase in the size of the network, from the egg-nauplius phase to the megalopa phase, and a centralization of the network around the node corresponding to the carapace. This node underwent a significant increase in size and color intensity. The betweenness centrality of the node corresponding to the carapace in the megalopa phase (15,880.5) was the highest reached by any node during the entire development.

On the other hand, the adult crab network underwent a radical structural topological reorganization. The node corresponding to the carapace lost priority and had a significant decrease in node degree and betweenness centrality. This led to a structural reorganization in which a node trinity took over. This node trinity concentrated a high betweenness centrality and consisted of the nodes corresponding to the fused thoracomere 1–4 (8,238.5), the cephalon (7,044) and the carapace (6,302.5). The betweenness centrality values of these nodes are denoted in parentheses.

Besides this triadic structure, the adult crab network was more compact than the one corresponding to the megalopa phase. This finding was supported by the aforementioned network parameters, where it was reported that the diameter, radius and average path length decreased in the transition from the megalopa to the adult crab. This was explained partially by the decrease in the size of the network (number of nodes). However, not entirely, as the decrease in the parameters mentioned (22.2 % in diameter and radius) was more pronounced than the decrease in size (17.1 %).

### Modularity

**Figure 4:**
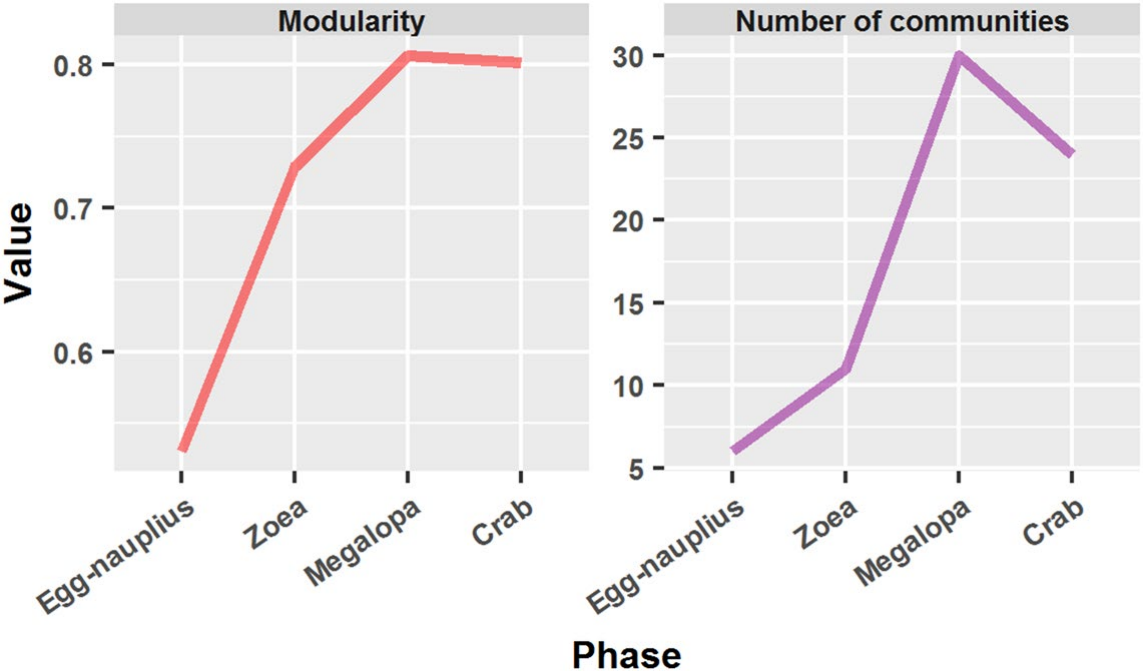
Modularity and number of communities in the networks corresponding to the different phases of crab development, obtained by multi-level optimization of modularity.

The method used for module detection and identification was multi-level optimization of modularity^45^. The networks representing crab metamorphosis resulted to be highly modular. Modularity increased during development from 0.530 in egg-nauplius to 0.807 in megalopa, with a slight decrease to 0.802 in the adult crab (Fig. 4). The number of communities also increased during development from 6 in egg-nauplius to 30 in megalopa, and decreased to 24 in the adult crab.

**Figure 5:**
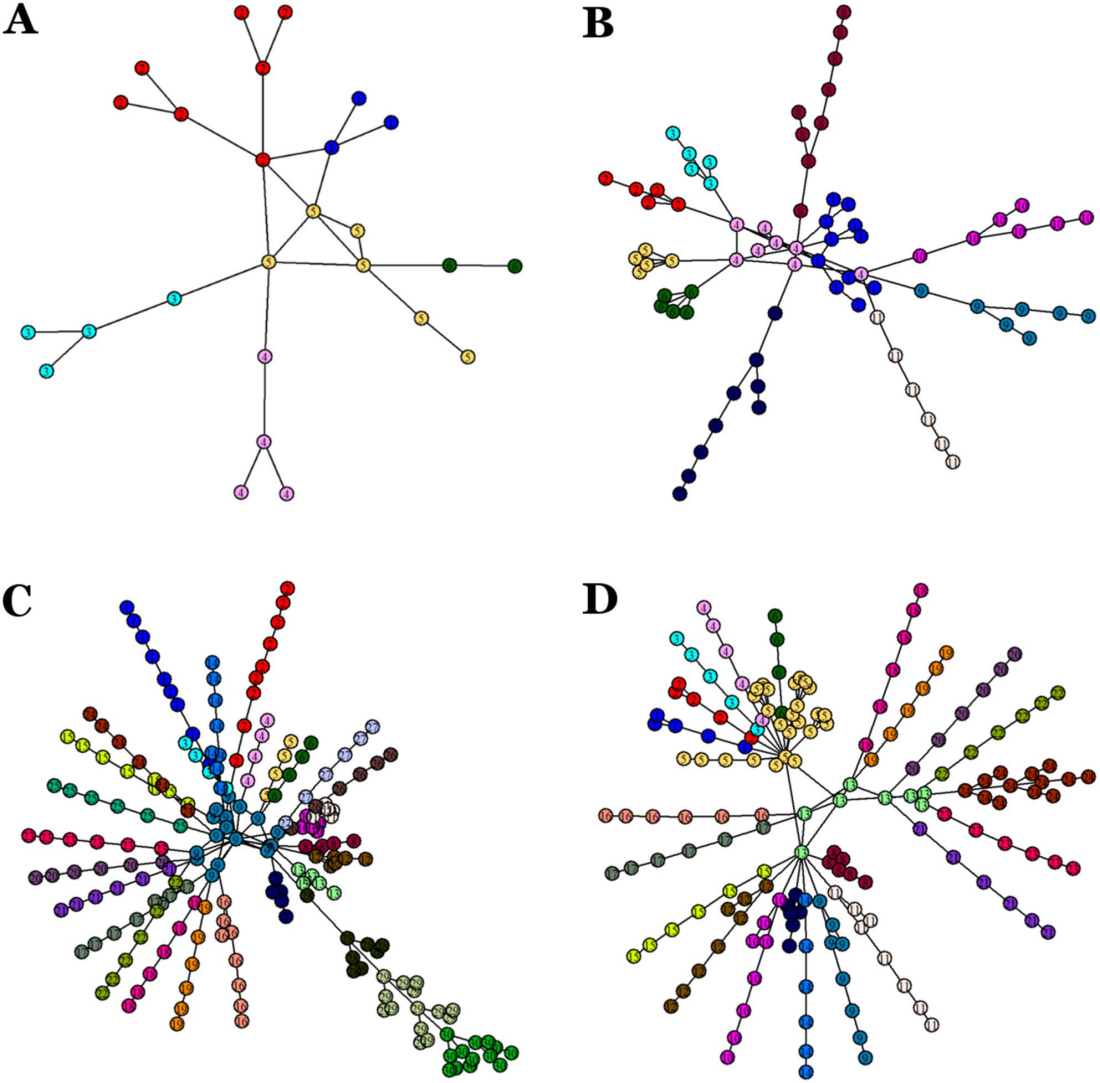
Module detection and identification in the different developmental networks: (**A**) Egg-nauplius, (**B**) Zoea, (**C**) Megalopa and (**D**) Crab (adult). Modules, obtained by multi-level optimization of modularity, are identified with different numbers and colors in the figure, and their composition detailed and specified in the accompanying table. Networks were plotted using the Fruchterman–Reingold layout algorithm.

The modules identified in each network are visualized and enumerated in Fig. 5 and Tables 2, 3, 4, and 5. As seen in our previous study^42^, each appendage was defined as a single module, with the exception of appendages consisting of few nodes, which were part of larger modules that included the body segments. In the egg-nauplius phase (Fig. 5A and Table 2), the first two body segments, carrying the eyes and antennule respectively, were defined as individual, rather simple, modules (module 1 and 2 in the figure). However, the rest of the body formed a single, more complex, module (module 5), held together and “hinged” by the node corresponding to the embryonic body. In the zoea phase (Fig. 5B and Table 3), three axial modules were detected which can be loosely associated with the concept of tagmata. The first one (module 1) consisted of the first three body segments with their corresponding appendages. The second one (module 4) consisted of the carapace, the other head segments, the two thoracomeres and the mandibles. The third one (module 11) comprised the abdomen, that is, the pleomeres and telson. In the megalopa phase (Fig. 5C and Table 4) this tagma-axial conformation is lost. There is no module separation between the head and the thorax, and the abdomen is subdivided in three contiguous axial modules, probably due to the presence of pleopods and uropods. Module 9 contained all the head and thoracic segments, gathered around and linked to the carapace. This evidenced the highly centralized structure of this network. Lastly, in the adult crab (Fig. 5D and Table 5) the previous (zoeal) “tagmatization” is restored. Module 5 contained the cephalon and most of its corresponding protruding appendages (except for the antennule, antennae and left mandible, which formed their own individual modules), and therefore could be interpreted as the head. Module 13 comprised all the thoracomeres (plus the penes) and could be regarded as the thorax. Finally, module 24 consisted of the pleomeres with its corresponding appendages, plus the telson, and therefore formed the abdomen.

**Table 2:** Modules in the network corresponding to the egg-nauplius phase.

Module	Membership
1	Head segment 1, eyes
2	Head segment 2, antennule
3	Right antenna
4	Left antenna
5	Embryonic body, head segment 3 and 4, right mandible, caudal papille
6	Left mandible

**Table 3:** Modules in the network corresponding to the zoea phase.

Module	Membership
1	Acron, head segment 1 and 2, eyes, antennule, antennae
2	Right maxillula
3	Left maxillula
4	Carapace, head segment 3 to 5, mandibles, thoracomere 1 and 2
5	Right maxilla
6	Left maxilla
7	Right first maxilliped
8	Left first maxilliped
9	Right second maxilliped
10	Left second maxilliped
11	Pleomere 1 to 5, telson

**Table 4:** Modules in the network corresponding to the megalopa phase.

Module	Membership
1	Right antennula
2	Left antennula
3	Right antenna
4	Left antenna
5	Right mandible
6	Left mandible
7	Right maxillula
8	Left maxillula
9	Carapace, head segment 1 to 6, eyes, thoracomere 1 to 8
10	Right maxilla
11	Left maxilla
12	Right first maxilliped
13	Left first maxilliped
14	Right second maxilliped
15	Left second maxilliped
16	Right third maxilliped
17	Left third maxilliped
18	Right first pereopod
19	Left first pereopod
20	Right second pereopod
21	Left second pereopod
22	Right third pereopod
23	Left third pereopod
24	Right fourth pereopod
25	Left fourth pereopod
26	Right fifth pereopod
27	Left fifth pereopod
28	Pleomere 1 and 2, second pleopods
29	Pleomere 3 and 4, third pleopods, fourth pleopods
30	Pleomere 5 and 6, fifth pleopods, uropods, telson

### ZP space: node roles within a module

The ZP space was divided in 7 regions^42^. Nodes with $P < 0.4$ were regarded as non-connectors, while nodes with $P \ge 0.4$ were considered connectors. Connectors with $P \ge 0.75$ were defined as hyper-connectors. Nodes with $z < 1$ were regarded as non-hubs, while nodes with $z \ge 1$ were considered hubs. Hubs with $z \ge 2.75$ were defined as super-hubs. Thus, the 7 defined regions were: 1) peripheral nodes (lightblue region, $P < 0.4$ and $z < 1$); 2) local hubs (pink region, $P < 0.4$ and $1 \le z < 2.75$); 3) local super-hubs (violet region, $P < 0.4$ and $z \ge 2.75$); 4) non-hub connector nodes (yellow region, $0.4 \le P < 0.75$ and $z < 1$); 5) non-hub hyper-connectors nodes (green region, $P \ge 0.75$ and $z < 1$); 6) connector hubs (orange region, $P \ge 0.4$ and $1 \le z < 2.75$); and 7) connector super-hubs (red region, $P \ge 0.4$ and $z \ge 2.75$).

**Table 5:** Modules in the network corresponding to the adult crab.

Module	Membership
1	Right antennula
2	Left antennula
3	Right antenna
4	Left antenna
5	Cephalon, eyes, right mandible, maxillule, maxillae
6	Left mandible
7	Right first maxilliped
8	Left first maxilliped
9	Right second maxilliped
10	Left second maxilliped
11	Right third maxilliped
12	Left third maxilliped
13	Carapace, thoracomere 1–4 (fused), thoracomeres 5 to 8, penes
14	Right first pereopod
15	Left first pereopod
16	Right second pereopod
17	Left second pereopod
18	Right third pereopod
19	Left third pereopod
20	Right fourth pereopod
21	Left fourth pereopod
22	Right fifth pereopod
23	Left fifth pereopod
24	Pleomeres 1 and 2, pleomere 3-5 (fused), pleomere 6, first gonopods, second gonopods, telson

**Figure 6:**
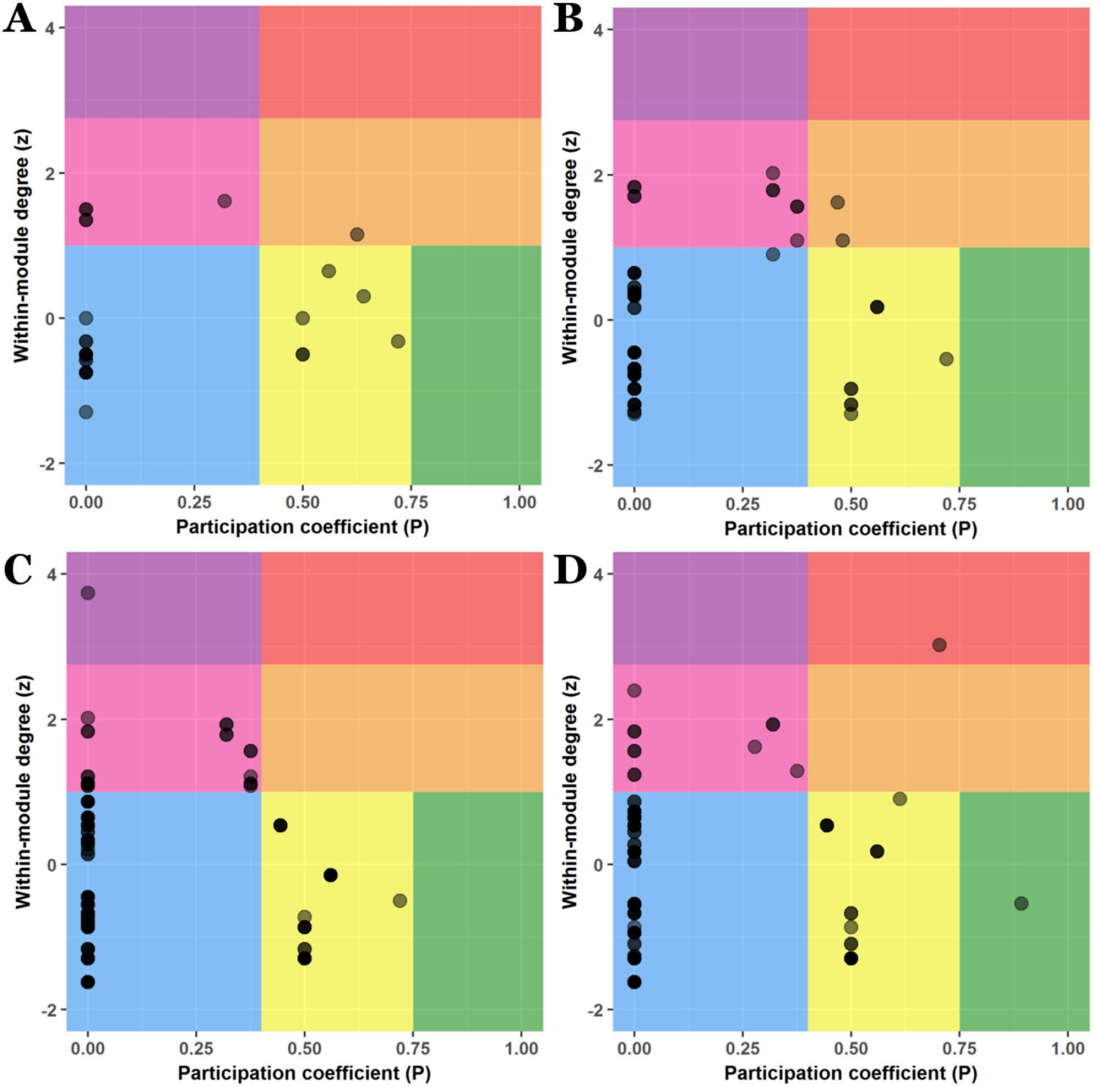
ZP space analysis. (**A**) Egg-nauplius, (**B**) Zoea, (**C**) Megalopa and (**D**) Crab (adult). See text for details and explanation of the different regions defined for the analysis.

In the egg-nauplius network, half of the nodes (53.85 %) were peripheral nodes (Fig. 6A). The rest of the nodes were non-hub connectors (23.08 %, including the embryonic body) or local hubs (19.23 %). The most distinctive characteristic of this network was the presence of a connector hub (orange region), represented by the first head segment.

In the zoea network, the percentage of peripheral nodes increased to 72.73 % (Fig. 6B). Also, the rest of the nodes were non-hub connectors (11.69 %) or local hubs (12.99 %). The most distintive characteristic of this network was the presence of 2 connector hubs (orange region): the second head segment and the carapace.

In the megalopa network, 73.33 % of nodes were peripheral nodes (Fig. 6C). The rest of the nodes were also non-hub connectors (16.19 %) or local hubs (10 %), with a greater number within the former region. However, a unique novelty was detected in this network: the presence of a local super-hub (violet region), represented by the carapace.

Finally, in the adult crab network, 79.31 % of nodes were peripheral nodes (Fig. 6D). The second most populated region was the one corresponding to non-hub connectors (13.22 %), and in the third place the region corresponding to local hubs (6.32 %, including the carapace). Most importantly, two unique novelties appeared in this network. In the first place, it was detected the presence of a non-hub hyper-connector (green region), represented by the fused thoracomere 1–4. In the second place, it was detected the presence of a connector super-hub (red region), represented by the cephalon.

In consequence, this analysis revealed very important features that will be key and crucial for the posterior analyses of hierarchy and complexity. In this analysis it could be revealed that the carapace fulfilled very different roles during development, passing from being a non-hub connector node in the egg-nauplius (simply the embryonic body at this phase) to a connector hub node in the zoea, a local super-hub in the megalopa and, finally, a local hub in the adult crab. Therefore, it passed from a rather non-hub connector role to a more local hub role. It seemed that its growing centrality as a hub increased at the expense of having a more and more restrictive and local activity and influence. However, in its last metamorphosis from megalopa to adult crab, the carapace lost a certain degree of centrality and preponderance, by losing its status of super-hub, and provoking a profound structural transformation in the network by the formation of a non-hub hyper-connector and a connector super-hub. The cause of this structural and topological transformation was to be found mainly in the fusion of head and thoracic segments, which occasioned the formation of the cephalon and fused thoracomere 1–4 structures.

### Hierarchical organization in crab developmental networks

The crab ontogenetic network series resulted to be highly modular, reaching values of modularity of 0.8 in the two final phases of development. But how are these modules organized in the overall topological structure of the network? In order to shed some light on this matter, I performed a topological overlap analysis^49^.

**Figure 7:**
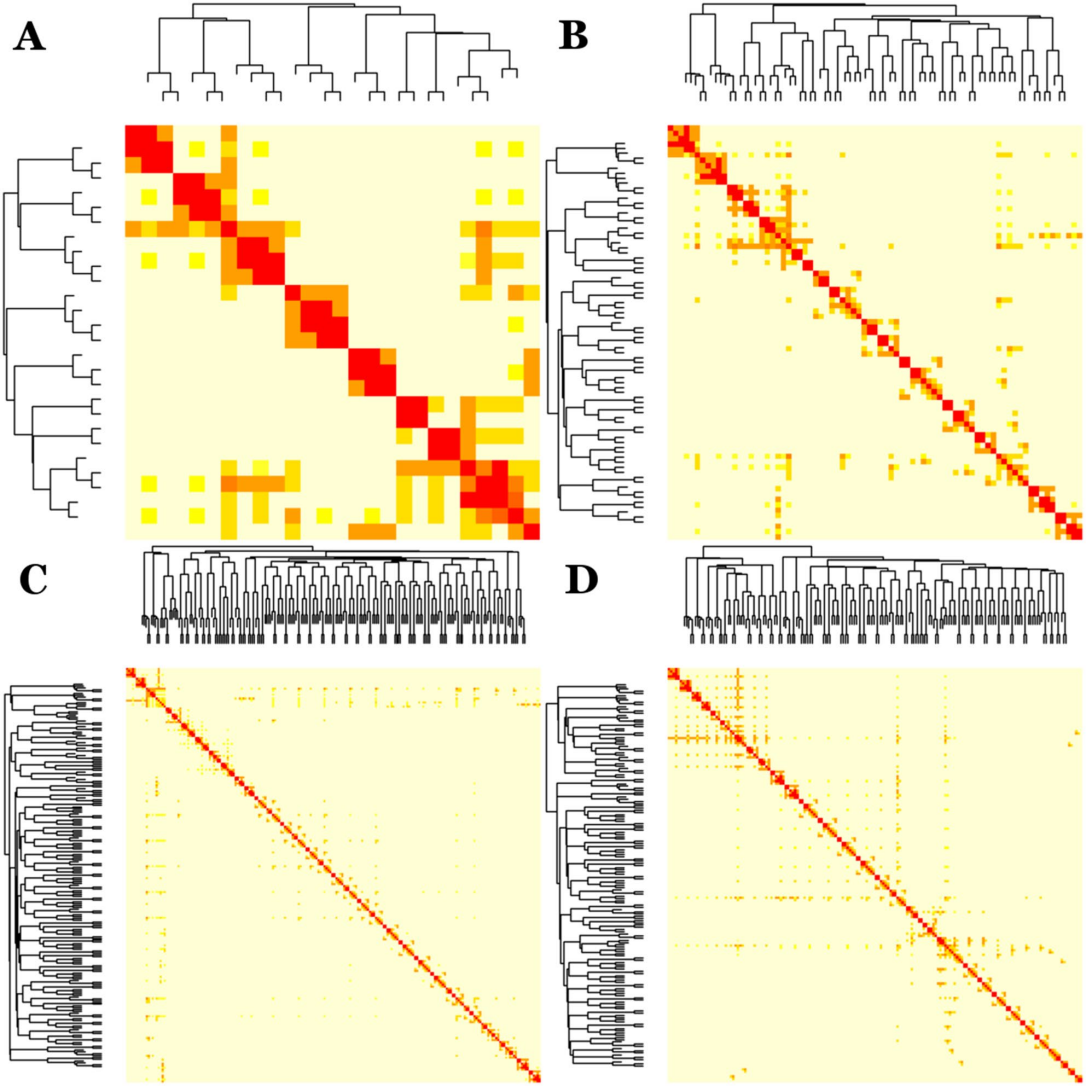
Topological overlap analysis: hierarchical organization of crab developmental networks. (**A**) Egg-nauplius, (**B**) Zoea, (**C**) Megalopa and (**D**) Crab (adult). The heatmaps represents the different topological overlap matrices. Rows and columns correspond to individual nodes, light colors represent low topological overlap, whereas orange and red colors represent progressively higher topological overlap. The corresponding hierarchical trees are shown on the left and top.

The topological overlap analysis showed that the networks have a hierarchical modular organization (Fig. 7). As seen in our previous study^42^, these networks presented two characteristic features: a modular organization and a module-within-module topological structure. The topological overlap matrix consisted of a series of blocks of high topological overlapping that were part of larger blocks, which is evidence of hierarchical modular organization. Two types of hierarchical organization could be detected: local and global. The first one was manifested by the module-within-module structure working at a short range. The second one was manifested by the presence of a wing-like structure extending over long ranges. Both types of hierarchical organization were present, with some differences, in all the ontogenetic network series.

In the egg-nauplius phase, 7 clearly demarcated, red-colored, blocks with high topological overlap were detected (Fig. 7A). The two modules at both extremes were forming a larger module (local hierarchical organization), being this topological overlapping more clear and intense in the case of the module at the bottom right. This module also exerted an influence that extended over nearly all the matrix (global hierarchical organization).

In the zoea phase, 13 blocks could be detected which possessed both submodules within and were also part of larger modules (Fig. 7B). The global hierarchical organization was not so evident than in the previous phase, but the third block starting from the bottom exerted a mild or moderate influence over the rest of the matrix.

In the megalopa phase, 34 blocks were serially disposed over the matrix diagonal, some of which contained submodules and some being part of larger modules (Fig. 7C). The most paradigmatic example of the latter was the third and the fourth blocks starting from the top, which formed a larger module that exerted a mild influence over the rest of the matrix. Some blocks in the middle possessed a middle range hierarchical organization, evidenced by the appearance of rather short wing-like structures.

Finally, in the adult crab, 32 blocks were detected forming, once more, submodules or supra-modules (Fig. 7D). The most striking characteristic of this topological overlap matrix was the abundant and intense presence of global hierarchical organization. The most intense (red-colored) wing-like structure was the one emitting from the seventh block from the top. The node responsible for this influence was the cephalon. The other two wing-like structures were less intense but of longer range. The nodes responsible for these global hierarchical organization structures were the fused thoracomere 1–4 and the carapace.

The inspection of the associated hierarchical trees confirmed the hierarchical organization of these networks. The number of levels of the hierarchical trees increased during development from 8 in the egg-nauplius phase, 11 in the zoea, to 13 in megalopa and adult crab. Despite this parity in the number of levels between megalopa and adult crab, these levels appeared much more well demarcated, that is, distributed more clearly at different heights, in the case of the adult crab. This fact, together with the results obtained with the topological overlap matrix, in which we saw more presence of global hierarchical organization in the adult crab, allowed us to conclude that this network was more hierarchical than the one corresponding to the megalopa phase. This conclusion will be very important when we analyze network complexity below.

### Complexity

#### Complexity measures

There is still an ongoing debate on what is a complex network. Some researchers consider that the most complex network is the complete network, whereas others consider it very simple: all nodes have nearly the same degree and there are no elaborate modularity structures. The latter think that a complex network is a network with modular structures at different levels and a medium number of links^51^. I tested these newly proposed complexity measures on the brachyuran ontogenetic network series. These measures possess the advantage of being normalized so that comparison between networks with different number of nodes, such as this case, is possible.

**Figure 8:**
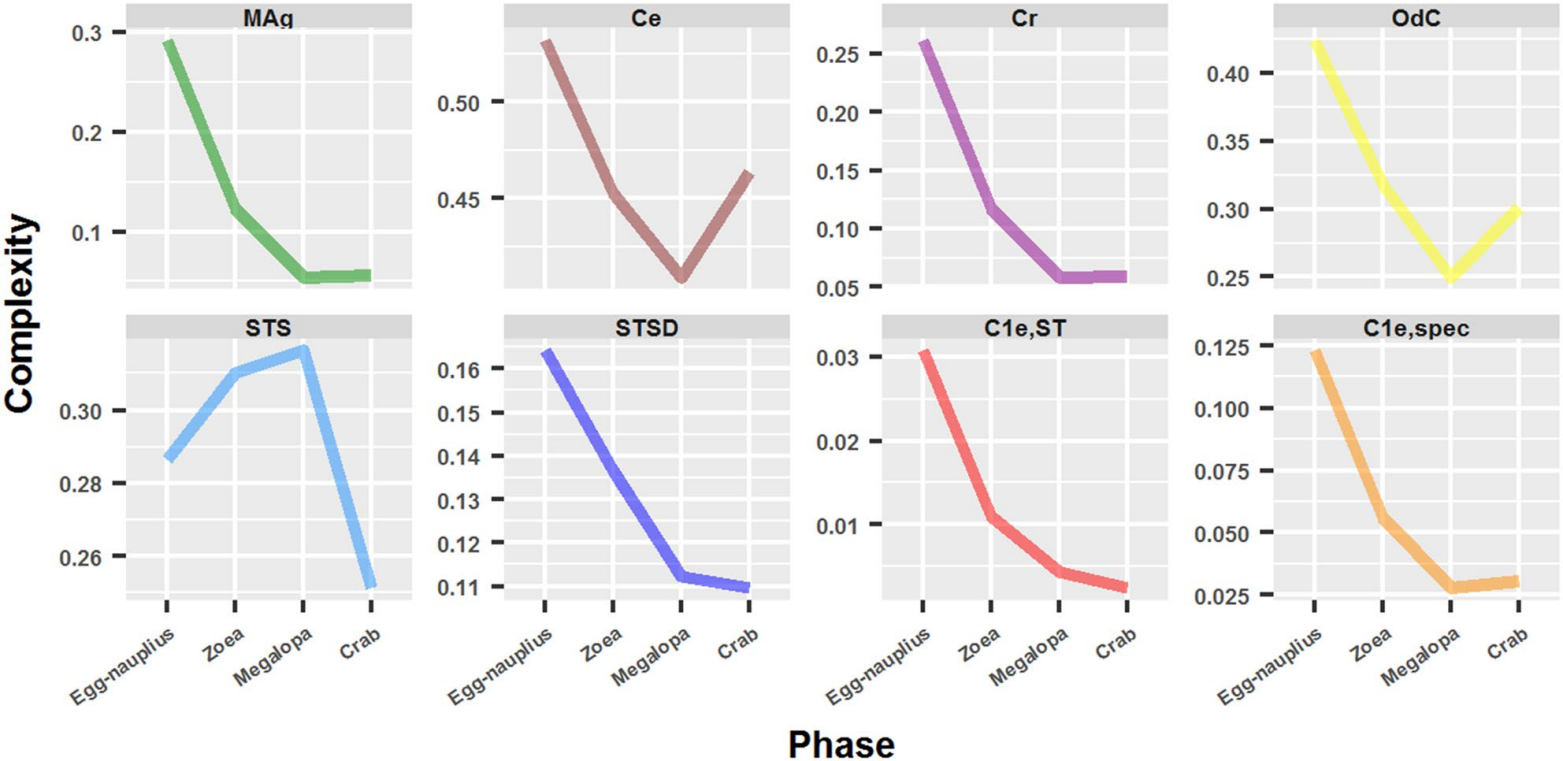
Complexity measures: their variation during crab development.

The results obtained were surprising and unexpected: most of the complexity measures tested decreased during development (Fig. 8). This was both strange and remarkable, since by inspecting Fig. 3 one could affirm almost without hesitation that network complexity increased during development, surely from the egg-nauplius to the megalopa phase, leaving the adult crab for further and deep consideration. Conversely, all the complexity measures tested decreased from the egg-nauplius to the megalopa phase, except for one: the Spanning Tree Sensitivity (*STS*), which registered a 10.4 % increment. All the other measures dropped, and most abruptly, such as $C_{1e,ST}$ (86.1 %), *MAg* (81.4 %), *Cr* (77.6 %) and $C_{1e,spec}$ (77.4 %), whereas the others diminished moderately or less severely, such as *OdC* (41.1 %), *STSD* (31.7 %) and *Ce* (23 %). In most cases, the transition from the megalopa to the adult crab did not imply a significant change in complexity. Within the exceptions we could count *Ce* and *OdC*, which registered a reversal and an increase of 13.2 % and 20.5 %, respectively. The inverse behavior in *STS* persisted in this final transition and registered a fall of 20.5 %.

#### Topological descriptors

Classical network parameters, such as average degree, average clustering coefficient, average path length and density, can be used to analyze complex networks, but their scope and reach is limited. There exists a battery of more sophisticated topological descriptors, some of which use information theory^54–56^. These measures enable the quantification of network structural information, their topology and metrical properties, and as such they represent true structural complexity measures.

**Figure 9:**
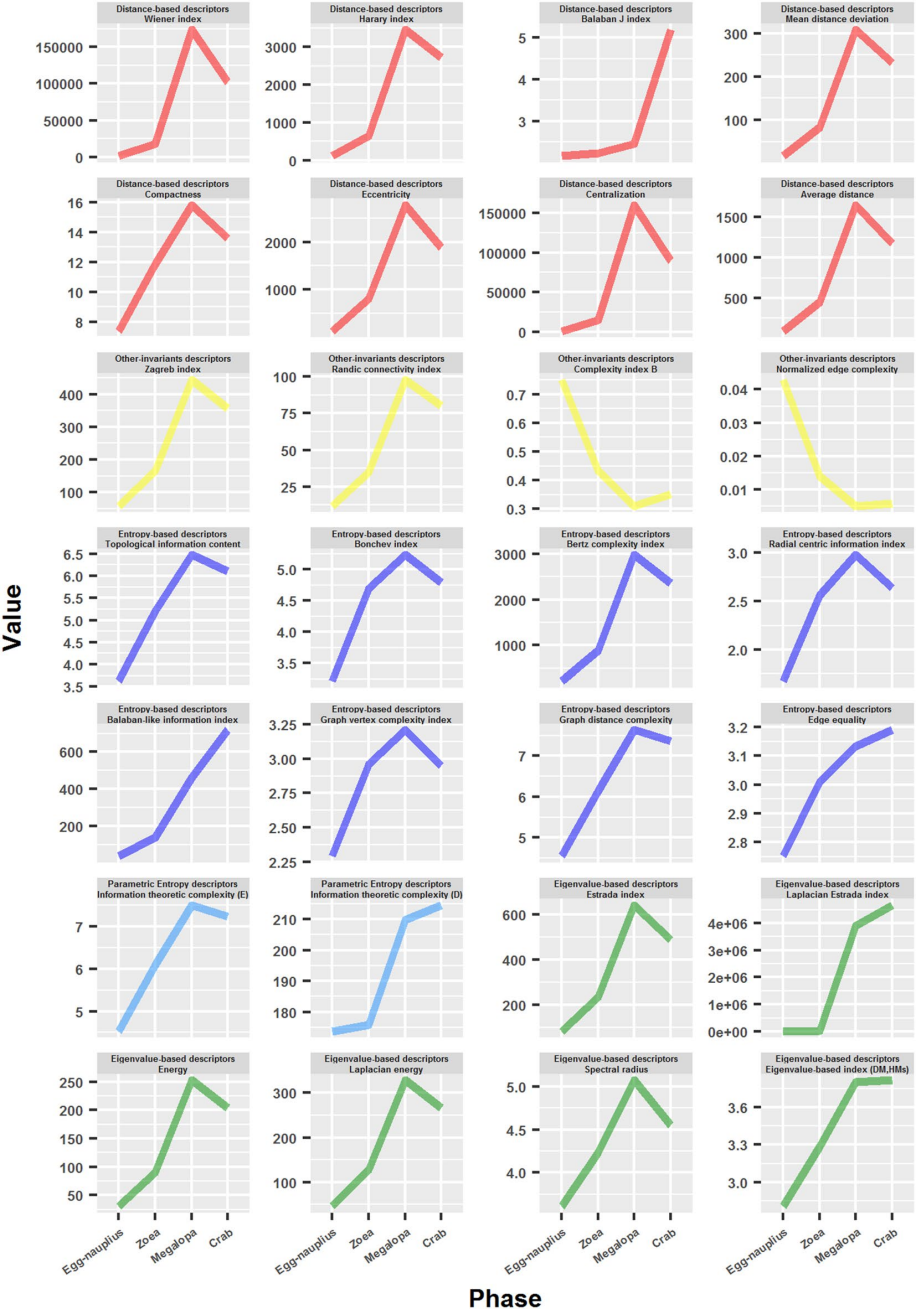
Topological descriptors: their variation during crab development.

Contrary to what happened with the previous complexity measures, the great majority of the topological descriptors increased during development (Fig. 9). There were only two exceptions: Complexity index B and Normalized edge complexity, which behaved very similarly to the previous complexity measures. The most important variability was reported, as usual, in the transition from megalopa to the adult crab. In various cases, it was registered a decline, whereas in others a further increase. This fact represented another key evidence for the occurring of a deep structural metamorphosis in the adult crab.

The most dramatic increase between the egg-nauplius and the megalopa network was reported in the Laplacian Estrada index (1,490.2 times), followed from afar by Centralization (175.2 times) and the Wiener index (144.8 times). In the majority of cases, the most important leap occurred in the transition from the zoea to the megalopa phase. Notable exceptions to this occurred, for example, with the Bonchev index and the Radial centric information index, in which the major leap was detected in the transition from the egg-nauplius to the zoea phase. On the other hand, there was one case in which the major leap occurred in the transition from megalopa to the adult crab: the Balaban J index. The other topological descriptors that also increased in this final metamorphosis were: Balaban-like information index, Edge equality, Information theoretic complexity (distance) and Laplacian Estrada index. As occurred with the complexity measures *OdC* and *Ce*, there was a reversal and increase in the Complexity index B at this final transition, although slighter than in the other cases.

#### Comparison of crab developmental networks with network models of similar characteristics

In order to compare the crab developmental networks with networks of similar characteristics, and to verify the existence of intrinsic and specific properties of them, 100 network models were generated from the network of each developmental phase and several of the parameters used in this work were tested. These network models not only had the same size (number of nodes), connection density (number of edges), but also the same degree sequence as the corresponding crab developmental network.

**Figure 10:**
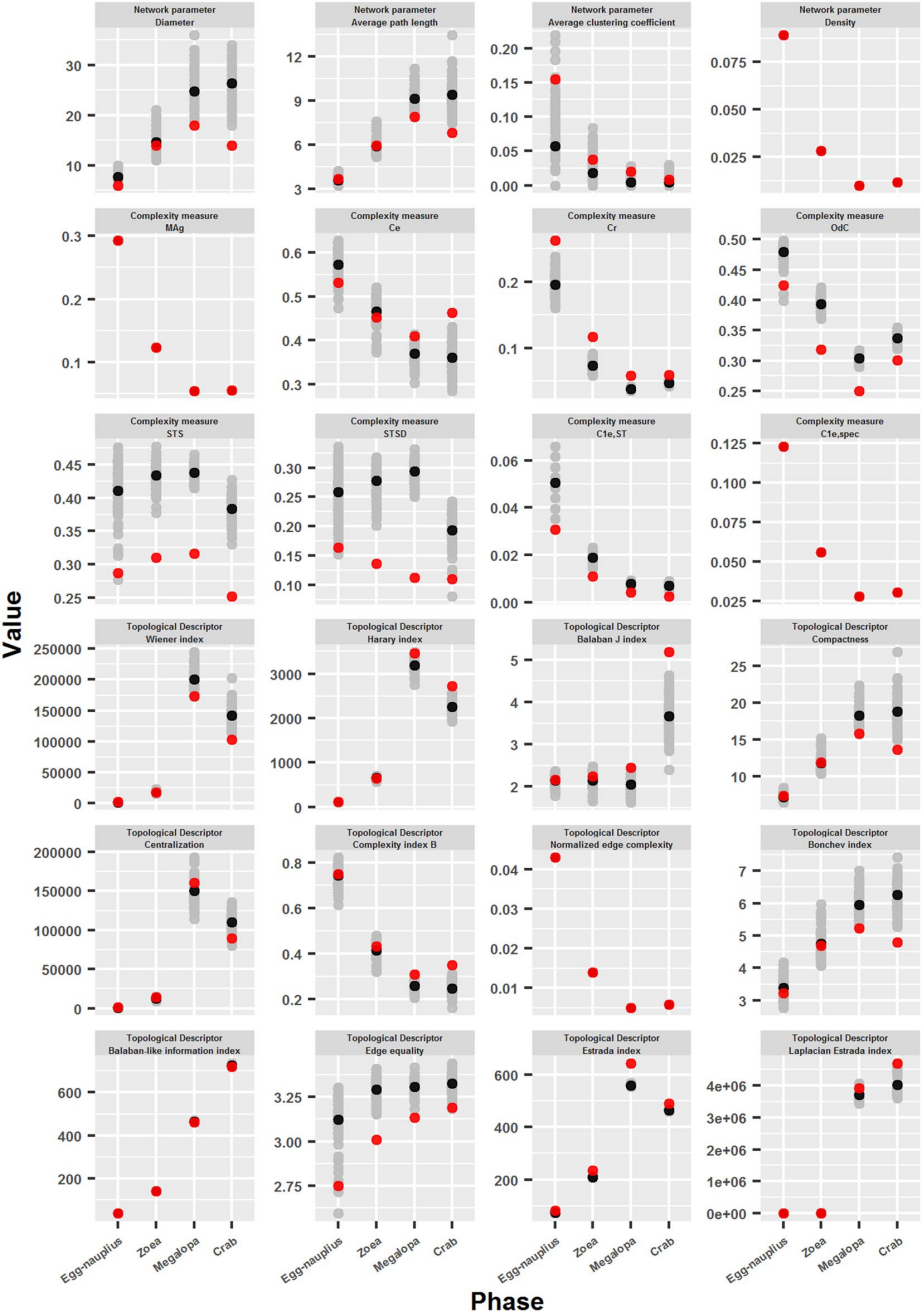
Comparison of crab developmental networks with network models of similar characteristics. 100 network models were generated from the network of each developmental phase, with equal size, connection density and degree sequence. The figure shows the values obtained for different parameters used in this work for the crab developmental networks (red points), the network models (gray points), and their mean value (black points).

In general terms, the network models (gray points, mean value: black points) had a similar behavior to the crab developmental networks (red points) (Fig. 10). Even some parameters, such as Density, *MAg*, $C_{1e,spec}$ and Normalized edge complexity were the same for all the generated networks, which confirms that these networks have very similar properties to each other and to the crab developmental networks, since they all have the same degree sequence. However, a great variation was seen in the values of several of the parameters measured, especially in the last stages of development (megalopa and adult crab). This was particularly evident in Diameter, Average path length, Average clustering coefficient, *Ce*, *STS*, *STSD*, $C_{1e,ST}$, Balaban J index, Compactness, Centralization, Bonchev index and Edge equality. Another peculiarity is that the crab developmental networks obtained substantially lower values than the network models for some parameters: especially *STS* and *STSD*, but also *OdC* and Edge equality.

**Figure 11:**
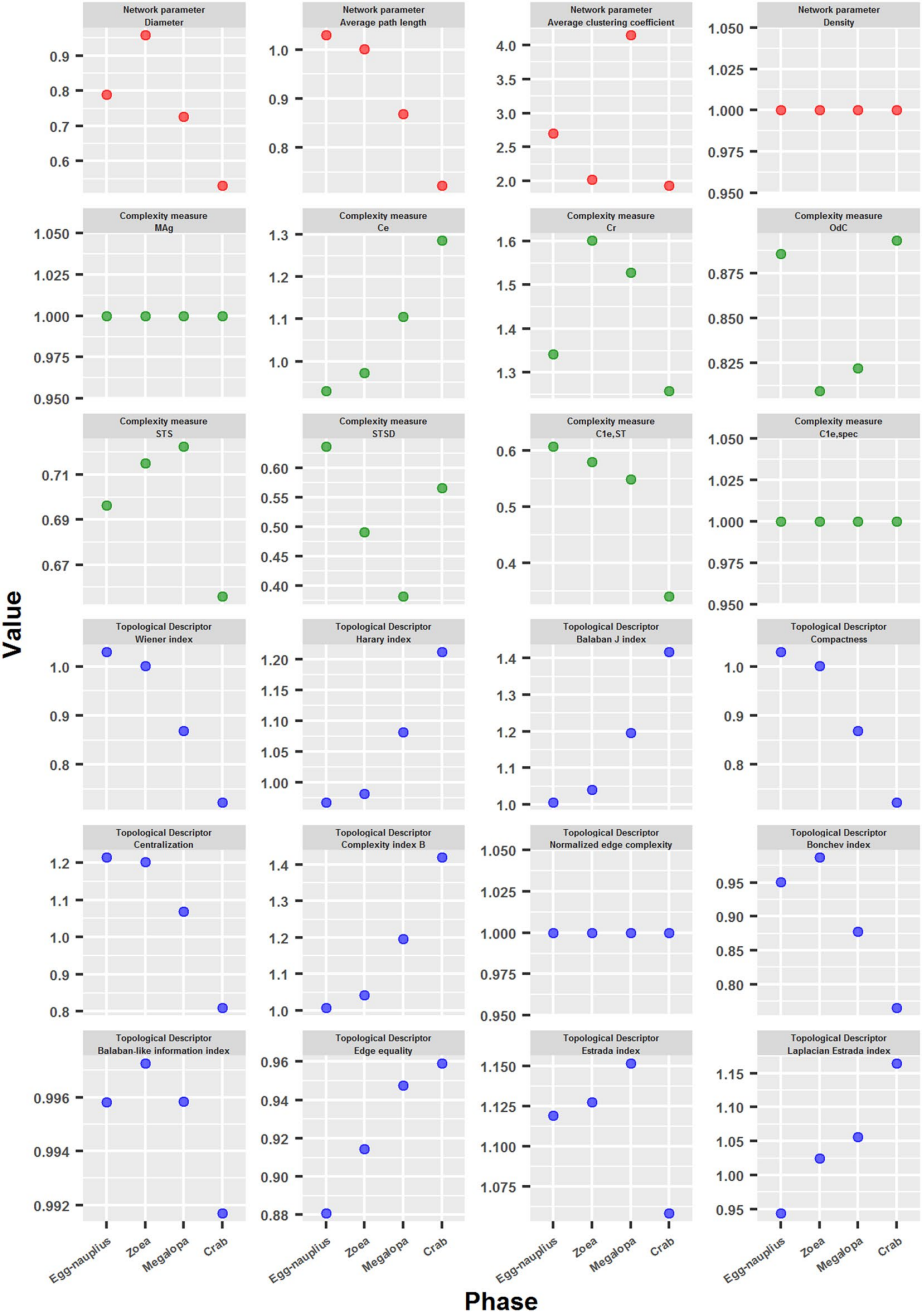
Comparison of crab developmental networks with network models of similar characteristics. The figure shows the quotients between the values obtained for the crab developmental networks and the mean value of the network models.

Crab developmental networks appear to have better performance for several of the measured parameters, especially for the later stages of development (megalopa and adult crab). This is evidenced in the lower values obtained in Diameter, Average path length, Compactness, Centralization, Wiener index and Bonchev index, and in the higher values obtained in *Ce*, Harary index, Balaban J index, Complexity index B, Estrada index and Laplacian Estrada index (see Fig. 11, values greater than 1 indicate that the network corresponding to the crab developmental phase has a value greater than the mean value of the network models). These results indicate that crab developmental networks are more compact, less centralized, more integrated and efficient than their respective models. Furthermore, the difference between the values obtained for the crab developmental networks and their respective models showed a clear tendency to increase in the successive phases of development, with the adult crab phase being the extreme case of this tendency. This was reflected in the obtaining of descending curves in the first group of parameters, and ascending curves in the second group of parameters (Fig. 11). The exception to this behavior was the Estrada index ratio obtained by the adult crab network, which was the lowest obtained in all developmental phases. This result, added to the previous ones, point to a radical metamorphosis of the topological structure of the adult crab network, going from a centralized, not very compact and efficient structure in megalopa, to an integrated, compact and more efficient structure in the adult crab.

### Vulnerability, protection and controllability

**Figure 12:**
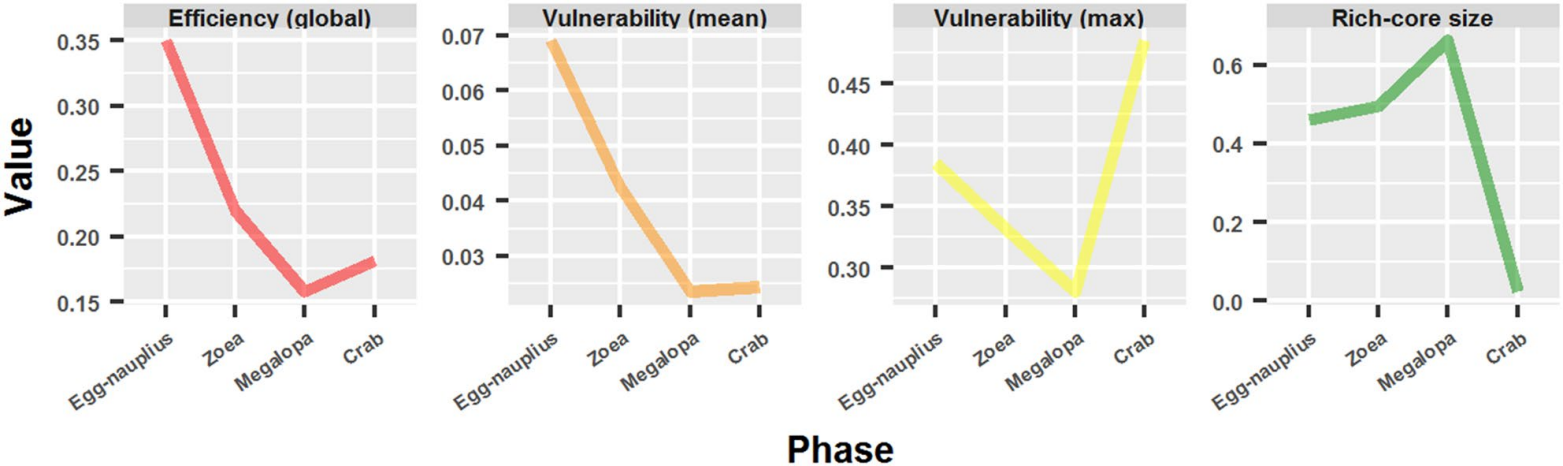
Vulnerability, protection and controllability in crab developmental networks.

The global efficiency^52^ decreased from the egg-nauplius to the megalopa phase, whereas it was reported an increase in the global efficiency from the megalopa to the adult crab network (Fig. 12). A similar behavior was found in the mean network vulnerability^83^. In this manner, the drop in the global efficiency was accompanied by a concomitant drop in the mean vulnerability. In comparison to megalopa, the adult crab network had a higher global efficiency with a minor or negligible alteration in the mean vulnerability. Unexpectedly, the maximum vulnerability increased drastically in the adult crab, even surpassing the value obtained for the egg-nauplius network. This behavior was adjudicated to the presence of two hyper-vulnerable nodes in the adult crab network, which corresponded to the cephalon (0.464) and the fused thoracomere 1–4 (0.485). However, from another point of view, the megalopa network had more nodes with a vulnerability superior to 0.1: 11 (5.2 %) against 7 (4 %).

Another reason could be revealed for the aforementioned increase of the maximum vulnerability in the adult crab network. A rich-core analysis was performed over the ontogenetic network series. This analysis revealed that from egg-nauplius (0.46) to megalopa (0.66) the rich-core size increased (Fig. 12). The final metamorphosis to the adult crab resulted in a dramatic change in the rich-core size (0.02), consisting of only 4 nodes (being 139 nodes in the previous phase). This radical transformation meant a transition from a highly flexible and adaptable network to a highly controllable network^73^.

### Error and attack tolerance

An error and attack tolerance test was carried out by removing nodes of the networks (randomly or selectively, respectively) and measuring their respective loss of connectivity^74^.

**Figure 13:**
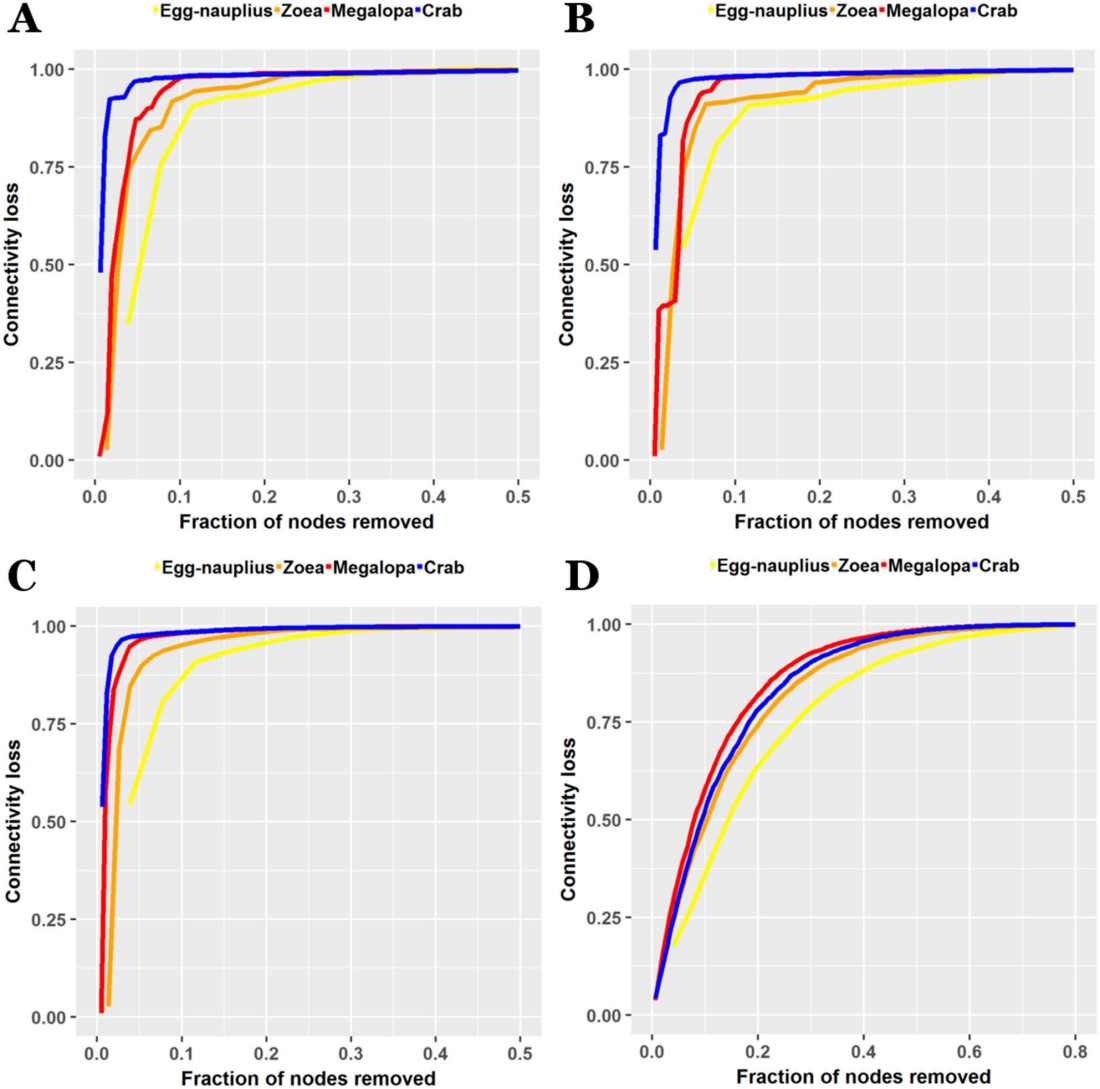
Error and attack tolerance analysis. Loss of connectivity due to the removal of an increasing fraction of nodes. Nodes are removed following different criteria: (**A**) in a decreasing order of their degree; (**B**) in a decreasing order of their betweenness; (**C**) using a cascading scenario, where betweenness are recalculated after each node is removed; (**D**) randomly.

**Figure 14:**
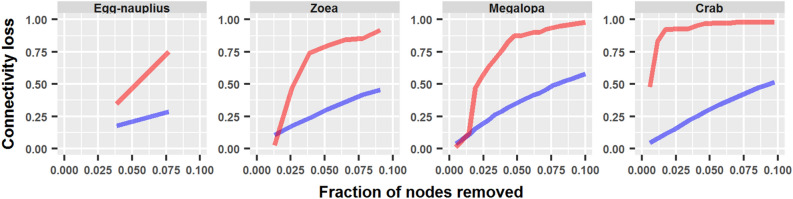
Error and attack tolerance during crab development. The difference between error (random, blue) and attack (degree, red) tolerance increased during development. It can also be visualized that error tolerance in the adult crab was higher (lower connectivity loss) than in the megalopa and zoea up to fraction of 0.033 of nodes removed.

Development produced an important decrease in tolerance against errors and attacks (Fig. 13). Moreover, the distance between both curves increased through development, reflecting an increasing imbalance between error and attack tolerance (Fig. 14).

However, a peculiar behavior occurred that disrupted this tendency. The adult crab network acquired a better tolerance to errors with respect to the megalopa network, and even the zoea network for a low fraction of nodes removed (approximately up to 3.3 %). In this manner, the adult crab network became very susceptible to attacks, but at the same time became more tolerant to errors than its ontogenetic predecessors.

## Discussion

### Crab development: structural topological network analysis

#### The transition from egg-nauplius to megalopa

The first steps in crab network development were marked by an evident increase in size and centralization. The number of nodes from egg-nauplius to megalopa increased from 26 to 210 (Table 1), that is, 8 times higher. This was accompanied by an increase in centralization around the node corresponding to the carapace, evidenced as an increase in node size and color in Fig. 3. However, this centralization was not conducive to a higher integration, that is, a global centralization, but, conversely, it condemned this hub to an increasing localization and short-range influence. This was evidenced by the transition of the carapace node role from a connector hub in the zoea phase into a local super-hub in the megalopa (Fig. 6). That in the transition from egg-nauplius to megalopa this increase in centralization did not mean an increase in the network integration, was also evidenced by the important increase in modularity (52.3 %, Fig. 4), passing from 6 to 30 modules.

On the other hand, network hierarchical organization seemed to lose predominance in the transition from zoea to megalopa (Fig. 7). Hierarchical organization was especially detected in egg-nauplius and zoea. In these phases, module-within-module structures were present, which are indicative of different levels of hierarchical organization. However, this nested organization was largely lost in megalopa, surely due to the predominant and hoarding role played by the carapace in this phase. Whereas the levels of the hierarchical trees increased from zoea (11) to megalopa (13), these levels were tightly packed in the latter while they were more clearly demarcated in the former, suggesting a clearer hierarchical organization in zoea.

#### The structural reorganization from megalopa to adult crab

In the final metamorphosis from megalopa to adult crab, two events occurred that produced a dramatic structural reorganization in the network: the fusion of the head segments 1 to 6, forming the cephalon, and the fusion of the thoracic segments 1 to 4, leading to the fused thoracomere 1–4. This resulted in the passage from a one-hub centralized network to a triadic distributed network organization. This triadic structure determined the appearance of two novel roles in the network: a connector super-hub (cephalon) and a non-hub hyper-connector (fused thoracomere 1–4). Meanwhile, the carapace lost preponderance and its role was reduced to a local hub. This topological reorganization produced a more compact and less centralized structure, as evidenced by the decrease in network diameter and average path length (Fig. 2), and the drop in compactness and centralization (Fig. 9; the lower the value of compactness, the more compact the network is). In other words, the adult crab network acquired a more integrated structure. The integration of this network was also manifested in its high hierarchical organization, evident both in the topological overlap matrix and its accompanying hierarchical tree (Fig. 7). This integration was secured and guaranteed by the two node novelties, the fused thoracomere 1–4 and the cephalon, especially the former acting as a hyper-connector.

The dramatic rise of the Balaban J index seemed to be another consequence of this structural reorganization. This index increased 111.45 % after this final metamorphosis (Fig. 9). This index increases with network size and branching^58^, so the index rised in spite of the network size reduction, therefore indicating a high increase in branching. This rise also supports the idea of a higher hierarchical organization in the adult crab network.

Another very interesting result was obtained with the Estrada index and the Laplacian Estrada index. Both indices increased during the development from egg-nauplius to megalopa, but whereas the Estrada index decreased in the final metamorphosis from megalopa to adult crab, the Laplacian Estrada index further increased. The Estrada index is known to be a network centrality measure^84^, so these results agree with the ones obtained with the centralization topological descriptor, cataloging the megalopa network as the one with the highest centrality. The Laplacian Estrada index, on the other hand, is considered to be, as a first approximation, a measure of branching^85^. The Laplacian spectrum appears to be a natural tool for the study of the expanding properties of networks^86^. Expanders are sparse networks with high connectivity properties, they are more robust and have more tolerance to errors. For example, regular networks are good expanders. It is postulated that the structural metamorphosis occurring at the transition from megalopa to adult crab, passing from a one-hub centralized network to a triadic structure topological network organization, is the responsible for the acquisition of a novel regular structure, absent in the previous phases of development, that makes the network more robust and tolerant to errors. This postulate was confirmed by the results obtained with the error and attack tolerance analysis, in which the adult crab network was more tolerant to errors than the corresponding megalopa network (Fig. 13).

The higher vulnerability to targeted attacks and the higher tolerance to errors in the adult crab network (Fig. 13) also allow us to extract conclusions regarding its structural topological organization. These results indicate that there are parts of the network (the most vulnerable to targeted attacks) that are more deeply integrated and sedimented in the whole organization. These structures will be more conserved, resilient and less prone to modification. At the same time, there are parts of the organization that are more superficially linked to the whole structure, i.e. the most tolerant to errors. Conversely, these parts could be easily changed and modified without a major damage to the overall organization. These different levels of sedimentation and stabilization present in the networks also favors the conclusion of a higher hierarchical organization in the adult crab network.

### Crab development as a process of unfolding of an intensive complexity

We saw that, in general terms, complexity measures decreased during development, whereas topological descriptors increased. The results obtained with the latter were the expected results. As development progressed, there was an evident increase in network size, distances and branching. The most typical case of this were the distance-based topological descriptors, such as the Wiener index, Harary index and Balaban J index.

On the other hand, the fall of complexity measures, such as *MAg*, *Cr* or *STSD*, was unexpected and surprising. These complexity measures are normalized measures that are based on different principles, they are product, entropy or subgraph measures. Product measures are information theoretic measures that are based on the idea that medium connectivity networks are beneficial and have better signal transmission, heritability and epistasis^51^. Therefore, they are defined as the product between how well they transmit information (i.e. short path lengths) and its “price” or “cost” (i.e. number of expensive links). For example, *MAg* is the product between mutual information and redundancy. Entropy measures quantify the *diversity* of different topological features. Monotonous networks are therefore considered as not complex. For example, *OdC* measures the diversity between a node of a given degree and neighbour nodes with higher degrees^53^. Finally, subgraph measures are based on the idea that a complex network is a network that contains many subgraphs^51^. For instance, $C_{1e,ST}$ quantifies the number of different spanning trees (i.e. the number of different subgraphs) after deleting one edge. It is counterintuitive and odd to accept that the egg-nauplius network contains more information at a low cost, a higher heterogeneity and a higher number of subgraphs, than the networks corresponding to the posterior developmental phases. In part, this effect may be explained by the fact that they are normalized complexity measures. But even so, it is still difficult to rationalize the overall behavior observed. It is clearly a property and behavior of a higher order, not explained by the complexity measures by themselves, but related to an inherent and characteristic property of the developmental process.

In view of the results obtained in this work, I propose the following postulates: (1) the existence of two kinds of complexity, intensive complexity and extensive complexity, and (2) that crab development implies the passage from an intensive complexity to an extensive complexity. Extensive complexity accounts for the actual complexity of the network, that is, the manifest, explicit, explicated and unfolded topological properties of the network, which in this work were measured by the topological descriptors. On the other hand, intensive complexity accounts for the potential or virtual complexity of the network, that is, the non-manifest, implicit, implicated and folded topological properties of the network, which in this work were measured by the complexity measures. In this manner, crab development consists of a process of unfolding from an intensive complexity into an extensive complexity, which coincides with the etymological meaning of the concept of evolution^3^. The extension of this postulate to development (and evolution) in general is a hypothesis that will be tested in future works. However, it is a hypothesis that becomes plausible if the theoretical considerations discussed in the next section are taken into account.

### A new look at development: preformation and epigenesis revisited

Development is now considered by many to be a process governed primarily by a genetic program. This genetic program is thought to be capable of generating form. This problem is already present in the historical dialectical conceptual tension between preformation and epigenesis. In all major epigenetic theory in history, development was considered to be a gradual process of organ formation starting from a homogeneous substance, and the agent responsible for generating the final form was a formative power. In this regard, the present genetic theory of development seems to be a more sophisticated form of the preformationist mosaic theory of development, in the sense that form seems to be guaranteed and attained by the positional effects generated by the genetic regulatory networks (GRNs). Instead of assuming a dissimilar division of the genetic material between daughter cells, it assumes a dissimilar pattern of expression between them. Consequently, this theory suffers from nearly the same problems as the theory developed by Roux and Weismann. Essentially, if all the (three-dimensional) information for the generation of the adult organism is already contained in the genetic material of the egg cell, how is this three-dimensional and positional information maintained and conserved after each cell division? The logical answer would be that it is not possible.

One way to circumvent this issue is to assume that the first cell divisions are indifferent, equal, and produce exact copies of the original egg cell, while the subsequent cells divisions are different, unequal and generate patterning. But, is this possible? Is a theory of development based on a material basis such as gene regulatory networks (composed of genes, transcription factors, proteins, etc.) capable of assuming and supporting an equal division of the egg cell? Either the egg cell is already unequally organized in different topological zones and gives rise to two different cells after the first division, or it is equally organized and does not possess different topological zones, which renders the theory useless. Here we can understand the ultimate conclusion of Driesch’s experiment: there is no material theory of development. His experiment shows in the most logical way that development cannot be explained materially. If the first two blastomeres can generate a whole animal, then there is no positional information contained in the egg cell and, therefore, its development cannot be explained by a structural and topological organization of a material substratum.

Edward De Robertis affirmed that “when an embryo is cut in half, it can self-regulate to regenerate the missing part”^87^. Although the terminology was misleading, he pointed out that the responsible for generating two embryos after separating the first two blastomeres was a process of “self-regulation”. Now, if “an embryo is cut in half”, can it “self-regulate to regenerate the missing part”? Certainly, in the strictest logical sense, if a part is lost, it cannot be regenerated. Somewhere, somehow, the information for its regeneration must persist. Regeneration, as well as development, always leads back to the fundamental tension between preformation and epigenesis. It also leads to consider how organismal form is attained from a single cell, and ultimately, from what is actually formless. Today, the question would be how organismal form is attained from a gene regulatory network (GRN), and if the GRN present in the egg cell can guide a developmental process that produces a functional and purposeful organism. The most fundamental question in this context would be if one can “arrive” at a form. That is to say, if form could be the result of a process of synthesis, if a whole can be obtained by the addition of a certain number of parts. This is what a true holism should address and answer. And the answer is that you can never obtain a whole through the addition of any number of parts, no matter how intricate they are organized: in order to obtain a true whole we must resort to the concept of form^88^. This leads us to consider the following: if form cannot be formed, that is, it cannot be made up or assembled, then it must exist from the beginning of the developmental process. Does this mean that the adult form is already present in the egg cell as in the old preformationist theories? Certainly not. It means that it has to exist, regardless of whether it is present or not, whether it is visible or not. This is what I mean when I affirm that a *virtual preformation* is necessary to explain development (and evolution)^3,4^, and that the concept of field may be important for that purpose^88^.

Therefore, form must be preformed, not actually, but virtually. This means that development would be a process of actualization of a virtual form. In other terms, it would be a process of unfolding of an intensive complexity. This work provided evidence of this process of actualization or unfolding in crab development using network theory. With this methodological framework, it was possible to reveal the passage from a virtual, potential, intensive complexity into an actual, extensive complexity. That is to say, it was possible to reveal what could be the fundamental process of what we call development.

## Supplementary information

**Supplementary information 1** Adjacency matrix corresponding to the egg-nauplius phase network.

**Supplementary information 2 **Adjacency matrix corresponding to the zoea phase network.

**Supplementary information 3** Adjacency matrix corresponding to the megalopa phase network.

**Supplementary information 4** Adjacency matrix corresponding to the adult crab phase network.
